# Expression of pluripotency markers in human granulosa cells after embryonic stem cell extract exposure and epigenetic modification

**Published:** 2012-05

**Authors:** Tahereh Talaei-Khozani, Ebrahim Kharazinejad, Laili Rohani, Zahra Vojdani, Zohreh Mostafavi Pour, Seyed Ziaadin Tabei

**Affiliations:** 1 *Department of Anatomy, Laboratory for Stem Cell Research, Shiraz University of Medical Sciences, Shiraz, Iran.*; 2 *Department of Biochemistry, Shiraz University of Medical Sciences, Shiraz, Iran.*; 3 *Transplantation Research Center, Shiraz University of Medical Sciences, Shiraz, Iran.*

**Keywords:** *Cell free extract*, *Embryonic stem cell*, *5-Aza-Deoxycytidine*, *Reprogramming*, *Trichostatin A*

## Abstract

**Background:** Epigenetic reprogramming of differentiated cells can modify somatic cells into pluripotential state. Pluripotency can be induced in somatic cells by several approches. One of the easy ways to induce pluripotency is the exposure of the somatic cells to the embryonic stem cell (ESC) extract.

**Objective:** The objective of this study was to increase the efficiency of reprogramming of granulosa cell as a differentiated cell into pluripotential state by using epigenetic modifier agents and extract.

**Materials and Methods:** The human granulosa cells were cultured in the medium containing 5-Aza-Deoxycytidine and trichostatin A. Then, the cells were exposed to mouse ESCs extract and co-cultured with mouse embryonic fibroblast in the presence of leukemia inhibitory factor (LIF). Alkaline phosphatase test and also immonohistochemistery staining for Oct4, Sox2 and Nanog were performed after 24 and 72 hours and 1 week.

**Results:** The granulosa cells showed the alkaline phosphatase activity after 24 hours and the enzyme activity maintained for 72 hours. They also expressed Oct4 after 24 hours. The cells also expressed Sox2 and Nanog, 72 hours after exposure to the ESCs extract. The expression of the pluripotency markers decreased after 1 week. It seems that the extract can induce dedifferentiation in granulosa cells and they can express the stem cell markers.

**Conclusion** **:** It seems that the inhibitors of the methyl transferase (5-Aza-Deoxycytidine) and histone deacetylase (trichostatin A) could delete the epigenetic markers and prepare the cells for reprogramming by administration of the extract.

## Introduction

Trans differentiation of differentiated somatic cells into other cell types is a promising approach for regenerative medicine. Reprogramming of the somatic cells was first shown by nuclear transfer technique in animal models (1). The research conducted by Takahashi and Yamanaka demonstrated the possibility of reprogramming somatic cells into a pluripotent stem cell state. It has also been shown that introduction of four factors such as Oct3/4, Sox2, Klf and c-Myc to fibroblasts could induce pluripotency (2). 

Gene expression pattern and epigenetic state of such induced reprogrammed stem cells are similar to those of ES cells (3). Pluripotency was also successfully induced via ectopic expression of four factors (Oct4, c-Myc, Sox2 and Klf) in human amniotic fluid-derived cells (4, 5), human embryonic fibroblast (6), mesenchymal stem cell and neural stem cell (7). 

It would be advantageous to induce pluripotency by methods that do not alter the genetic content of the cells. Reprogramming of the somatic cell lines by administration of cell free extract was done by some researchers (8-10). It has been shown that differentiated somatic cells such as fibroblasts could be dedifferentiated by exposing the cells to embryonic carcinoma cells (11) or ESCs extract (8-10). The nuclei of the mammalian cells can be partially reprogrammed into an embryonic state by Xenopus egg extracts (12). 

The expression of pluripotency markers such as Oct4 and alkaline phosphatase in fibroblasts and human primary leukocytes was also shown. These cells were exposed to the extract from Xenopus laevis eggs and early embryos (13). However, the rate of pluripotency markers expression, after extract treatment, took approximately twice as long when compared with retroviral reprogramming (14). It seems the factors that control pluripotency are very conservative and the transcription factors in the extract can reprogram the somatic cells of the other species. 

In a trans-differentiation process, the epigenetic markers should be deleted and various elements in the microenvironment should be allowed to make the new epigenetic markers. Using epigenetic modifier agents caused the cells to be reprogrammed more efficiently. For instance, using valporic acid, a histone deacetylase inhibitor, enables primary human fibroblasts to be reprogrammed by two transcription factors such as Oct4 and Sox2, without the need for the oncogenes *c-Myc* or *Klf4 *(15). 

Administration of 5-Aza-Deoxycytidine** (**5-Az-dc), inhibitor of DNA methyl transferase, has been shown to improve the efficiency of reprogramming process (16). Histone deacetylase inhibitor, trochostatin A (TSA), could also improve the epigenetic markers expression in early cloned bovine embryos (17). Reprogramming was done in different cell types. Fibroblasts and human primary leukocytes were reprogrammed into pluripotent state by administration of the extract (13). 

Granulosa cells have also been shown to be reprogrammed successfully by nuclear transfer technology (18, 19) and the embryos derived from fresh cumulus cells developed to term (20). Granulosa cells from human sources can be obtained during IVF procedure. Ovarian cells were also demonstrated to express pluripotency markers, when they were cultured in feeder in the presence of LIF. The granulosa cells lose their specific markers and could express pluripotency markers (21). 

However, the granulose cells expressed the stem cell factor (22); they did not express germ cell markers including Nanog, Vasa and Stellar (26). The hypomethylated chromatin in the cells treated with epigenetic modifier agents can be exposed to other pluripotency induced elements in the extract. These factors may reprogram the somatic cells. Therefore, the objectives of this study were to induce expression of pluripotency markers in the granulosa cells by extract exposure and to improve the efficiency of markers expression by epigenetic modification.

## Materials and methods

**Granulosa cell culture**

The study design is experimental intervention. The granulosa cells were obtained from the healthy patients who were involved in IVF programs and their husbands had infertility problem. The protocol was approved by the ethic committee of Shiraz University of Medical Sciences and the patients were volunteers and signed the consents. 

The granulosa cells were dissociated by trypsin/EDTA and washed once with culture media. The pellets were resuspended in DMEM/F12 containing 20% FBS, 1% L-glutamine and 1% penicillin/ streptomycin (all from Gibco). 

The cell culture with confluency of 50% was treated with 2µM of 5-Az-dc (Sigma) and 0.5µM of TSA (Sigma) for 24 hours. Two µM of 5-dAC was added for additional 72 hours (23). The vehicle was added to the granulosa cells as control. The culture media were changed every day. Cell viability was assessed by trypan blue (24). 

**Embryonic stem cell culture**

ESCs (R1 line) were cultured under embryonic condition in DMEM (Gibco) supplemented by 10% FCS, 1% L-glutamine, 1% non-essential amino acids, 0.1mM β mercaptoethanol (Sigma), 1000U leukemia inhibitory factor (LIF) (Chemocon) and 1% penicillin/streptomycin on a gelatin coated plate. Mouse embryonic fibroblasts were used as feeder layer deactivated by mitomycin (Sigma). 

**Extract preparation**

ESCs were isolated from the feeder layer by their various attachment times. The ESCs were harvested enzymatically by trypsin/ EDTA (Gibco) and were plated on gelatinized flask for one and half hour. The non-attached cells were harvested and washed with PBS without Ca and Mg (PBS^-^) two times. 

The cells were washed with Ca and Mg- free Hank's buffer salt solution (HBSS) once and resuspended in an equal volume of freshly prepared ice cold cell lysis buffer comprising of 50 mM NaCl, 5 mM MgCl_2_, 20 mM HEPES, pH 8.2, 1 mM dithiothreitol (DTT), 0.1 mM and protease inhibitor cocktail (Sigma), for 45 min. Then, the swelled cells were sonicated by pulse sonicators (Heilscher) until complete lyses of the cells as judged by light microscopy. The lysate was sedimented at 15000g for 15min at 4^o^C. The supernatant was collected, snap-frozen in liquid nitrogen (9) and were stored at -80^o^C until used. Total protein concentration was 5.34 mg/mL, being assessed by Bicinchonic acid/cooper sulfate test (BSA Protein Assay Kit, Pierce) as manufacture's instruction indicated. 

**Cell permeabilization and extract treatment**

Harvested granulosa cells were washed with cold PBS^-^ three times. The cells were resuspended in HBSS; cell counting was performed and aliquoted in 20000 cells in 16.4 µl of cold HBSS. The cells were incubated at 37^o^C for 2 minutes. 4.6 µL of streptolysin O (Sigma) at final concentration of 230ng/ml was added to the cells and then incubated at 37^o^C for 50 minutes. Twenty µL of the extract containing ATP regenerating system [ATP, GTP, creatine phosphate and creatine kinase (all from Sigma)] and 25mM of NTP (Sigma) were added to the cells, followed by incubation at 37^o^C for 1 hour.

Preheated culture media containing 2mM CaCl_2_ were added to the cells and then transferred to 48 well culture palates. The cells were allowed to attach to the culture plates for 2-4 hours. The culture media were replaced with DMEM containing 10% FCS, 1% penicillin/ streptomycin and 1% L glutamine1% non-essential amino acids, 0.1mM β mercaptoethanol, 10ng/mL LIF. The cells were cultured for 24, 72 hours and also 1 week (9). 

**Permeabilization assay**

To ensure the cells were permeablized effectively, premeabilization assay was done. The assay was based on the uptake of the FITC-conjugated 70 000 M_r _Dextran (Sigma) by premeablized cells. Uptake was detected with fluorescent microscopy (11). 

**Immunofluorescence**

The pluripotency markers were detected by anti-Oct4 (10µg/mL), anti-Nanog (10µg/ml) and-anti Sox2 antibody (at same dilution) (all from R & D). The cells were washed with PBS^-^ and fixed by 4% paraformaldehyde for 20 minutes. The fixed cells were washed and incubated in PBS^-^ containing 10% goat serum, 1% BSA and 1% triton X. 

The primary and FITC- conjugated secondary antibodies were used for 1 hour. The secondary antibody was gout FITC -conjugated anti-human antibody (Sigma) at a concentration of 1:500. The cells were mounted and observed by fluorescence microscopy (Nicon E600). 

**Alkaline phosphatase assay**

Alkaline phosphatase activity was assessed as a pluripotency marker by alkaline phosphatase assay kit (Sigma). The staining procedure was done according to the manufacturer's instruction. Briefly, the cells were washed with PBS and fixed in acetone/ formaldehyde/ citrate fixative solution for 30 seconds at room temperature. 

The cells were washed and incubated in alkaline phosphatase substrate containing Fast Red Violate, Naphtol AS-BI and sodium nitrate for 15 min. The cells were washed in deionized water and counterstained with hematoxylin.

## Results

**Granulosa cell culture**

**Figure 1:**
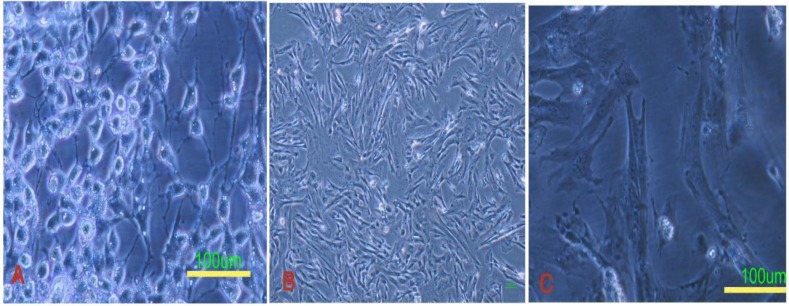
Granulosa cells before (A) and after (B, C) exposure to the trichostatin A and 5-deoxyazacytidine

The granulosa cells were attached to the plate 2-3 hours after platting. They displayed a fibroblast-like morphology (Figure 1A). After 2 passages, they were treated with the TSA and 5-Az-dc. The TSA induced not only the cell death but also changed the morphology of the cells that survived to more fusiform with fewer processes (Figure 1B). 5-aza-2-deoxycytidine did not influence the cell viability when it was administrated alone, as indicated by trypan blue assay. 

**Granulosa cell morphology after extract treatment**

**Figure 2:**
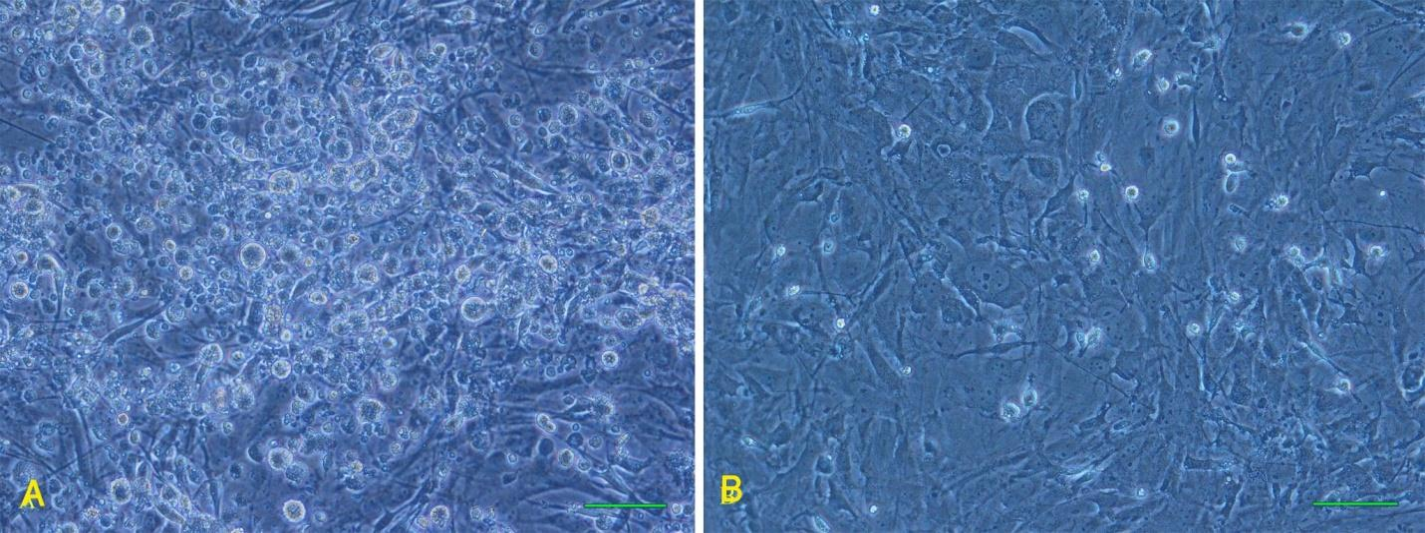
The granulosa cells after exposure with ESC extract (A). The cells formed rounded aggregates. The permeablized untreated cell formed very fewer aggregates (B). Scale bar is 100µm

**Figure 3:**
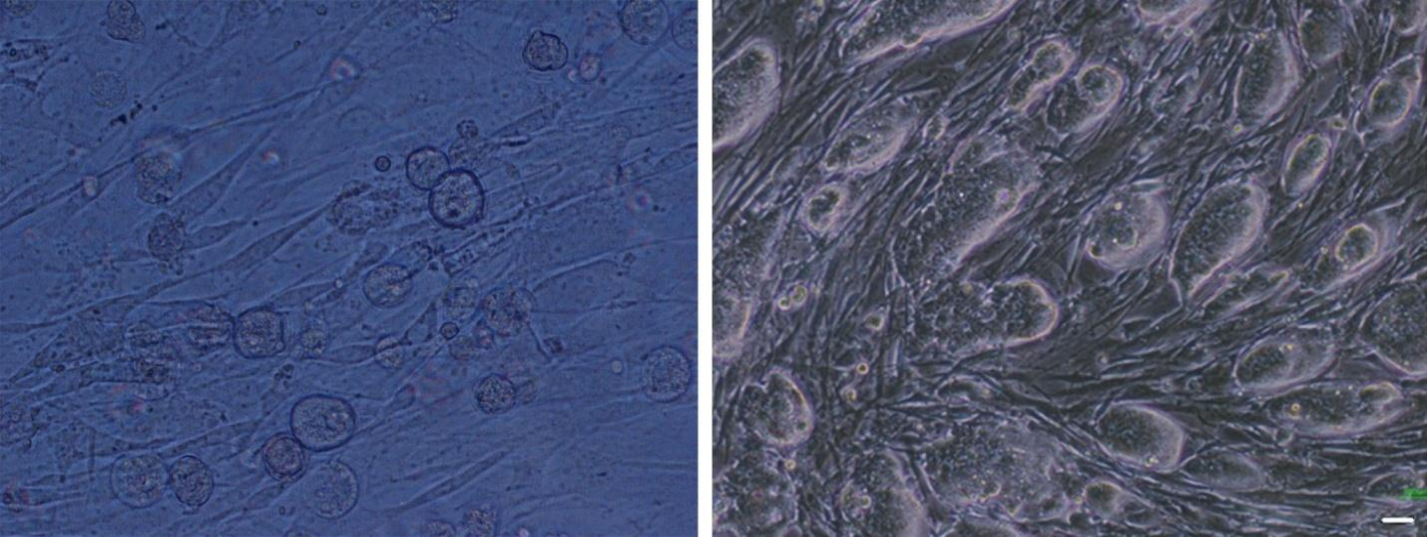
The granulosa cells were exposed to the ESC extract and then, cultured on mouse embryonic fibroblast cells as feeder (Left), and colony of the ESC cultured on mouse embryonic fibroblast cells (Right).

Forty-eight hours after treatment with ESCs extract, the granulosa cells formed the round cell aggregates. The number of these cell aggregates was more than those which were treated with chromatin modifier agents without cell extract administration (Figure 2). These cells were also cultured in the same condition as ESCs (LIF and feeder). Although the morphology of the cell aggregates was comparable with ESC colony, their size was smaller than ESC colony (Figure 3). 

**Alkaline phosphatase assay**

**Figure 4:**
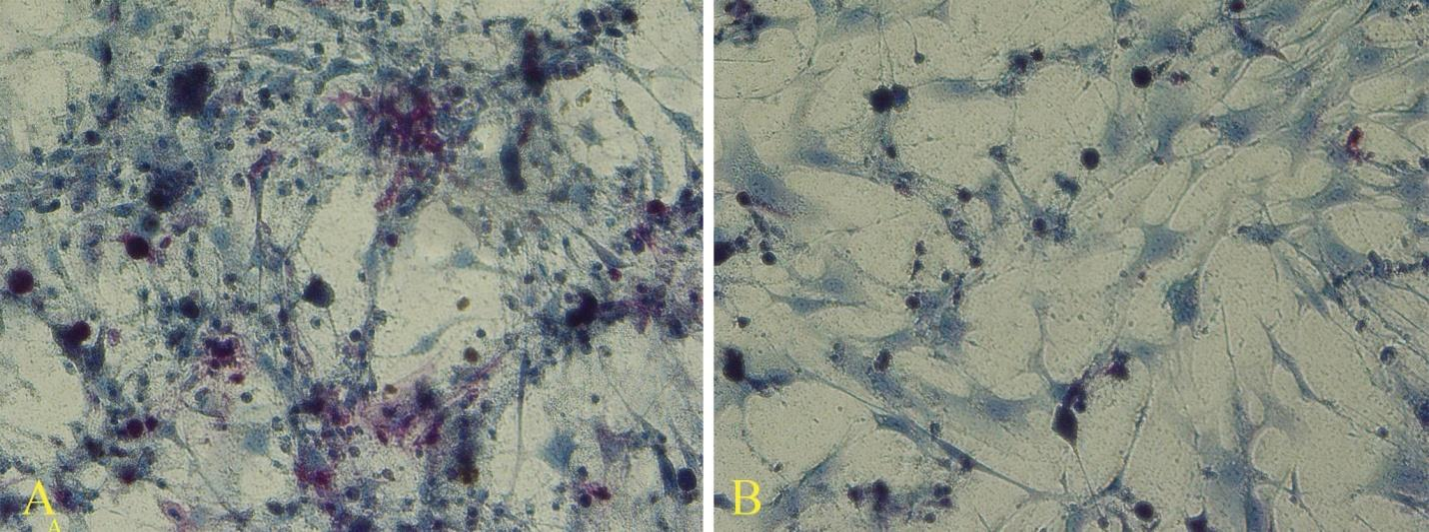
Alkaline phosphatase assay revealed cells treated with 5-aza-2-deoxycytidine and trichostatin A, in the presence of theextract expressed alkaline phosphatase (A). The number of the alkaline phosphatase positive cells was fewer when they exposed to the chromatin modifier agents without extract (B).

In cultures exposed to TSA and 5-Az-dc and permeabilized in the presence of ESCs extract, positive alkaline phosphatase activities were detected. Most of the cells expressing this enzyme gathered in rounded cell aggregates; however, the single cells with fibroblast-like morphology also showed positive reaction to alkaline phosphatase (Figure 4). In cultures exposed to chromatin modifier agents without any extract as well as the non-treated granulosa cells cultured in the presence of feeder and LIF, also showed the enzyme activity. 

**Figure 5:**
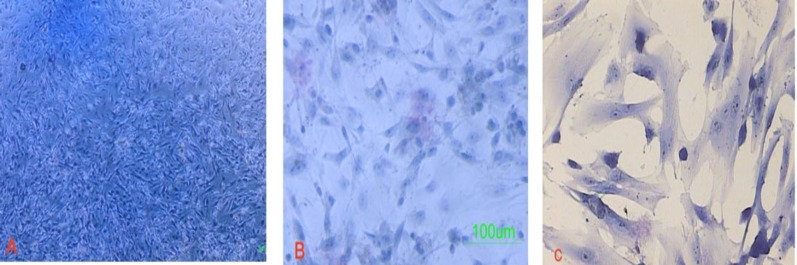
The non-treated granulosa cells that cultured in the presence of feeder and LIF (A). Higher magnification showed some cells with very weak alkaline phosphatase activity (B). The non-exposed cultures to the feeder or LIF did not express alkaline phosphatase (C).

However, the number of the cells that expressed the alkaline phosphatase was less than those exposed to the chromatin modifier agents and extract (Figure 5A, B). The granulosa cells that were not treated with the extract and also TSA and 5-Az-dc could not express alkaline phosphatase activity (Figure 5C). 

**Expression of pluripotency markers**

Immunocytochemimical assays showed a notable degree of Oct4 expression in granulosa cells that exposed to the TSA and 5-Az-dc and also ESC extract after 24 hours of incubation. These cells could not express Sox2 and Nanog at this time. The cells that were not treated with the extract could not express any of pluripotency markers after 24 hours of incubation in ESC culture condition. 

**Figure 6:**
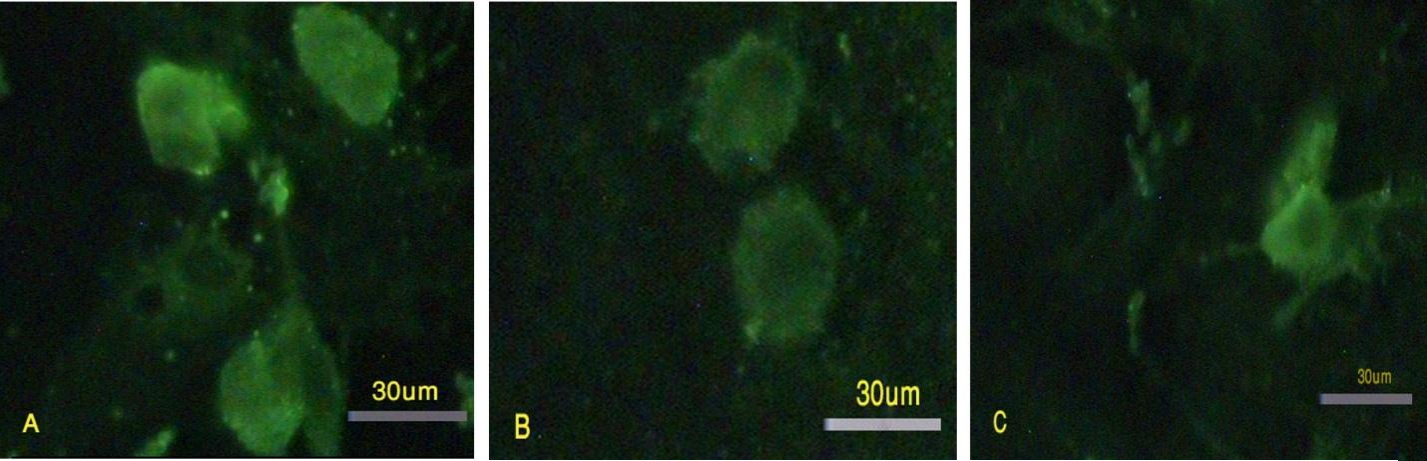
After 72 hours of incubation in the presence of LIF and feeder, the granulosa cells expressed Oct4 (A), Sox2 (B) and Nanog (C).

**Figure 7:**
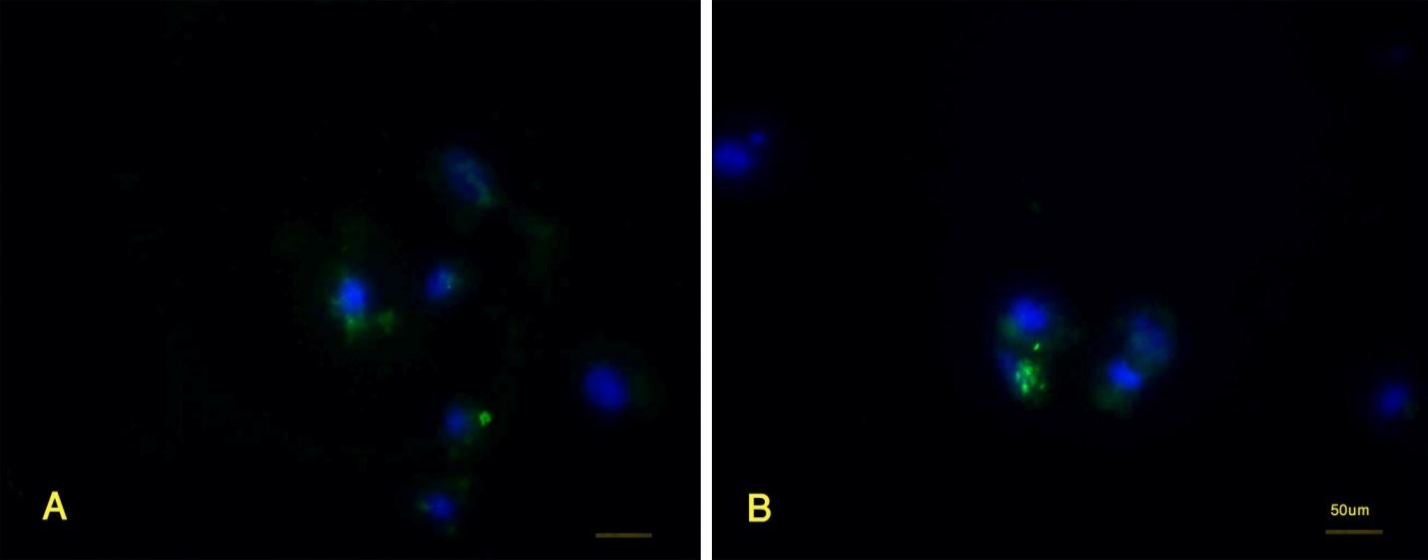
The granulosa cells that were culture in the presence of feeder and LIF (A) and those that were exposed to the trichostatin A and 5-azacytidin but not ESC extract (B) could also express Oct4 after 72 hours of incubation. However, the intensity of the reaction was less

After 72 hours, the chromatin modifier and extract treated-granulosa cells expressed Oct4, Sox2 and Nanog with high intensity (Figure 6). The granulosa cells that were cultured in the presence of feeder and LIF (Figure 7A) and those treated with TSA and 5-Az-dc without ESC extract and cultured in the presence of LIF (Figure 7B) could also express Oct4 after 72 hours of incubation; However, the intensity of the reaction decreased. These granulosa cells could not express Sox2 and Nanog. The granulosa cells cultured in DMEM/F12 could not express pluripotency markers. After 168 hours of incubation in ESCs culture condition, the granulosa cells lost the ability to express pluripotency markers. They could not express Oct4, Sox2 and Nanog. 

## Discussion

Experiments involving somatic nuclear transfer (1) and fusion of differentiated cells with ESCs (25) have shown that reprogramming could be performed in fully differentiated cells. Production of pluripotent stem cells without destruction of the oocyte is one of the goals in regenerative medicine (26). 

The first remarkable attempt for induction of pluripotency by extract was done by Taranger *et al* (2005). They showed that in the presence of the extract, fibroblasts expressed Oct4 and Alkaline phosphatase. As the result of Oct4 and Alkaline phosphatase expression, the reprogrammed cells were capable of forming embryoid like bodies (11). Incubation of fibroblastic cell line in the ESCs extract for 1 hr, resealing them by CaCl_2_ and continually culturing for weeks, caused ESCs specific markers expression. Various reports showed that the pluripotency markers are expressed at different period of times (6, 10, 11). Some pluripotency markers such as Sox2 were detected only after 8 weeks of culturing (10). Our data showed that administration of the TSA and 5-Az-dc induced the granulosa cells to express pluripotency markers such as Oct4 and alkaline phosphatase after 24 hours. This may be accelerated by administration of chromatin remodeling agents that deleted the epigenetic markers and prepared the cells to re-establish the new ones. 

However, the presence of these markers after 24 hours may be related to their existence in the ESCs extract that may enter into the cells during permeabilization, thus causing false positive (8). However, if it were true, it should have detected Sox2 and Nanog as well but it did not happen in this study. Besides, various ESC lines source for extract preparation and LIF concentration can influence the duration of the reprogramming process and the types of markers expressed after extract exposure (8). In this study, primary culture of human granulosa cells was chosen to induce pluripotency instead of fibroblast cell lines that were used in other studies (8, 11, 12).

Fibroblasts can be reprogrammed permanently and they can maintain their pluripotency at the presence of LIF (13). Reprogramming efficiency is significantly influenced by the cell type (4). This study showed the granulosa cells loss their ability to express pluripotency markers after 1 week. These may be related to one of the following phenomena: the differentiation of the cells that took place if the cells were not passaged, temporary reprogramming of the cells and the type of the cells used as donor. 

Luteinizing granulosa cells have been considered as terminally differentiated cells. These cells undergo death after ovulation (27). However, we found they could proliferate for more than 10 passages in vitro. It has been shown that these cells can be cultured for a prolonged time in the presence of LIF. Under this condition, the granulosa cells could lose their specific markers and express Oct4 but not the other pluripotent markers (27). Our data confirmed this finding. When non-treated granulosa cells were cultured in the presence of LIF and feeder, they could express Oct4 and also alkaline phosphatase. However, the expression of the pluripotency markers was down-regulated and the number of the cells that expressed these markers was few and the intensity of the reaction was very weak. 

Temporal expression of the ESC markers was also reported in other cell types such as corneal epithelial cells that pre-exposed to cell free ESC extract (28). It has been reported that the extract-derived iPS cells had different proteomes and global gene expression patterns compared with ESCs (29).

The genes that are responsible for pluripotency are regulated by epigenetic mechanisms involving DNA methylation and histone modifications (30). Epigenetic modification induces the cells to reprogram (31). 5-Aza-Deoxycytidine or TSA has been shown to increase most imprinted gene transcripts in ESCs. In this way, these agents guided ESCs to differentiate into specific cellular lineages (32). 

On the other hand, several stem cell and pluripotency-associated genes were expressed in the neurosphere cells that are induced by 5-Az-dc or TSA (33). It has been shown that treatment of 293T cells with ESCs extract for 1-8 hours modified histone on the Oct4 and Nanog promoters and then increased the expression of pluripotent marker genes (9). It has also been reported that the fibroblast cell line (NIH3T3) could express embryonic markers when they are pre-exposed to TSA and 5-dAZ (33). In this study, granulose cells expressed pluripotency markers after TSA and 5-dAZ treatment as well. 

However, the intensity of the reaction and the number of the positive cells were diminished, compared with the cells exposed to both chromatin modifier agents and extract. The administration of the chromatin modifier agents could prepare the pluripotent genes to response to the factors present in the ESCs extract. In our experimental model the combined administration of the extract and chromatin-modifier agents accelerated the expression of the pluripotency markers.

## Conclusion

In conclusion, the granulosa cells could express the pluripotency markers after ESCs extract exposure and in the presence of LIF. The efficiency could increase if the chromatin modifier agents were used before treatment with ESCs extract. However, they could not maintain the ability for a long time. 

## Footnote

***This article extracted from M.Sc. Degree thesis.***

## References

[1] Wilmut I, Schnieke AE, McWhir J, Kind AJ, Campbell KH (1997). Viable offspring derived from fetal and adult mammalian cells. Nature.

[2] Takahashi K, Yamanaka S (2006). Induction of Pluripotent Stem Cells from Mouse Embryonic and Adult Fibroblast Cultures by Defined Factors. Cell.

[3] Wernig M, Meissner A, Foreman R, Brambrink T, Ku M, Hochedlinger K (2007). In vitro reprogramming of fibroblasts into a pluripotent ES-cell-like state. Nature.

[4] Li C, Zhou J, Shi G, Ma Y, Yang Y, Gu J (2009). Pluripotency can be rapidly and efficiently induced in human amniotic fluid-derived cells. Hum Mol Genet.

[5] Zhao HX, Li Y, Jin HF, Xie L, Liu C, Jiang F (2010). Rapid and efficient reprogramming of human amnion-derived cells into pluripotency by three factors OCT4/ SOX2/ NANOG. Differentiation.

[6] Zhou W, Freed CR (2009). Adenoviral gene delivery can reprogram human fibroblasts to induced pluripotent stem cells. Stem Cells.

[7] Tat PA, Sumer H, Jones KL, Upton K, Verma PJ (2010). The efficient generation of induced pluripotent stems (iPS) cells from adult mouse adipose tissue-derived and neural stem cells. Cell Transplant.

[8] Neri T, Monti M, Rebuzzini P, Merico V, Garagna S, Redi CA (2007). Mouse fibroblasts are reprogrammed to Oct-4 and Rex-1 gene expression and alkaline phosphatase activity by embryonic stem cell extracts. Cloning Stem Cells.

[9] Bru T, Clarke C, McGrew MJ, Sang HM, Wilmut I, Blow JJ (2008). Rapid induction of pluripotency genes after exposure of human somatic cells to mouse ES cell extracts. Exp Cell Res.

[10] Xu YN, Guan N, Wang ZD, Shan ZY, Shen JL, Zhang QH (2009). Cell extract-induced expression of pluripotent factors in somatic cells. Anat Rec (Hoboken).

[11] Taranger CK, Noer A, Sørensen AL, Håkelien AM, Boquest AC, Collas P (2005). Induction of dedifferentiation, genomewide transcriptional programming, and epigenetic reprogramming by extracts of carcinoma and embryonic stem cells. Mol Biol Cell.

[12] Miyamoto K, Furusawa T, Ohnuki M, Goel S, Tokunaga T, Minami N (2007). Reprogramming events of mammalian somatic cells induced by Xenopus laevis egg extracts. Mol Reprod Dev.

[13] Hansis C, Barreto G, Maltry N, Niehrs C (2004). Nuclear reprogramming of human somatic cells by xenopus egg extract requires BRG1. Curr Biol.

[14] Cox JL, Rizzino A (2010). Induced pluripotent stem cells: what lies beyond the paradigm shift?. Exp Biol Med.

[15] Huangfu D, Osafune K, Maehr R, Guo W, Eijkelenboom A, Chen S (2008). Induction of pluripotent stem cells from primary human fibroblasts with only Oct4 and Sox2. Nat Biotechnol.

[16] Mikkelsen TS, Hanna J, Zhang X, Ku M, Wernig M, Schorderet P (2008). Dissecting direct reprogramming through integrative genomic analysis. Nature.

[17] Iager AE, Ragina NP, Ross PJ, Beyhan Z, Cunniff K, Rodriguez RM (2008). Trichostatin A improves histone acetylation in bovine somatic cell nuclear transfer early embryos. Cloning Stem Cells.

[18] Fujii W, Funahashi H (2008). In vitro development of non-enucleated rat oocytes following microinjection of a cumulus nucleus and chemical activation. Zygote.

[19] Sugawara A, Sugimura S, Hoshino Y, Sato E (2009). Development and spindle formation in rat somatic cell nuclear transfer (SCNT) embryos in vitro using porcine recipient oocytes. Zygote.

[20] Akagi S, Kaneyama K, Adachi N, Tsuneishi B, Matsukawa K, Watanabe S (2008). Bovine nuclear transfer using fresh cumulus cell nuclei and in vivo- or in vitro-matured cytoplasts. Cloning Stem Cells.

[21] Gong SP, Lee ST, Lee EJ, Kim DY, Lee G, Chi SG (2010). Embryonic stem cell-like cells established by culture of adult ovarian cells in mice. Fertil Steril.

[22] Høyer PE, Byskov AG, Møllgård K (2005). Stem cell factor and c-Kit in human primordial germ cells and fetal ovaries. Mol Cell Endocrinol.

[23] Koh E, Bandle R, Clair T, Roberts DD, Stracke ML (2007). Trichostatin A and 5-aza-20-deoxycytidine switch S1P from an inhibitor to a stimulator of motility through epigenetic regulation of S1P receptors. Cancer Lett.

[24] Freshney RI (2005). Culture of animal cells, a manual of basic technique.

[25] Do JT, Schöler HR (2010). Cell fusion-induced reprogramming. Methods Mol Biol.

[26] Rajasingh J, Lambers E, Hamada H, Bord E, Thorne T, Goukassian I (2008). Cell-free embryonic stem cell extract-mediated derivation of multipotent stem cells from NIH3T3 fibroblasts for functional and anatomical ischemic tissue repair. Circ Res.

[27] Kossowska-Tomaszczuk K, De Geyter C, De Geyter M, Martin I, Holzgreve W, Scherberich A (2009). The multipotency of luteinizing granulosa cells collected from mature ovarian follicles. Stem Cells.

[28] Zhan W, Liu Z, Liu Y, Ke Q, Ding Y, Lu X (2010). Modulation of rabbit corneal epithelial cells fate using embryonic stem cell extract. Mol Vis.

[29] Hattori N, Imao Y, Nishino K, Hattori N, Ohgane J, Yagi S (2007). Epigenetic regulation of Nanog gene in embryonic stem and trophoblast stem cells. Genes Cells.

[30] Kim YJ, Ahn KS, Kim M, Shim H (2011). Comparison of potency between histone deacetylase inhibitors trichostatin A and valproic acid on enhancing in vitro development of porcine somatic cell nuclear transfer embryos. In Vitro Cell Dev Biol Anim.

[31] Baqir S, Smith LC (2006). Inhibitors of histone deacetylases and DNA methyltransferases alter imprinted gene regulation in embryonic stem cells. Cloning Stem Cells.

[32] Ruau D, Ensenat-Waser R, Dinger TC, Vallabhapurapu DS, Rolletschek A, Hacker C (2008). Pluripotency associated genes are reactivated by chromatin-modifying agents in neurosphere cells. Stem Cells.

[33] Zhang XM, Li QM, Su DJ, Wang N, Shan ZY, Jin LH (2010). RA induces the neural-like cells generated from epigenetic modified NIH/3T3 cells. Mol Biol Rep.

